# Laparoscopic resection of a torted ovarian dermoid cyst

**DOI:** 10.1186/1749-7922-2-12

**Published:** 2007-05-09

**Authors:** Katie M Williams, Charles J Bain, Michael D Kelly

**Affiliations:** 1 Department of Laparoscopic and Upper GI Surgery, Frenchay Hospital, Bristol, UK

## Abstract

Torsion or rupture of an ovarian cyst may present as an acute abdomen. A case is presented where the diagnosis was made at laparoscopy and laparoscopic resection was done. Controlled aspiration of the cyst contents allowed the cyst to be easily removed from the abdomen.

## Background

Torsion or rupture of an ovarian cyst may present as an acute abdomen. Sometimes the diagnosis will only be made at laparoscopy and this can be unsettling for a non-gynecologic surgeon. We present such a case and discuss our management.

## Case presentation

A 20 year old young woman presented to the emergency department with a sudden onset of severe right iliac fossa pain of 12 hours duration. The pain was constant with no radiation and was rapidly worsening prior to her admission. It was associated with anorexia and vomiting. On direct questioning there were no relevant gynecoligic or urologic symptoms. The patient reported a similar, but milder episode, one month ago when she was seen at another hospital. No diagnosis was made at the time and the pain resolved after analgesia.

On examination, she was haemodynamically stable and apyrexial. She was tender in the right iliac fossa with guarding and rebound tenderness. Hematological blood tests revealed a neutrophilia and a urinary pregnancy test was negative. The patient was listed for an urgent laparoscopy with the provisional diagnosis of appendicitis or ruptured ovarian cyst.

**Figure 1:**
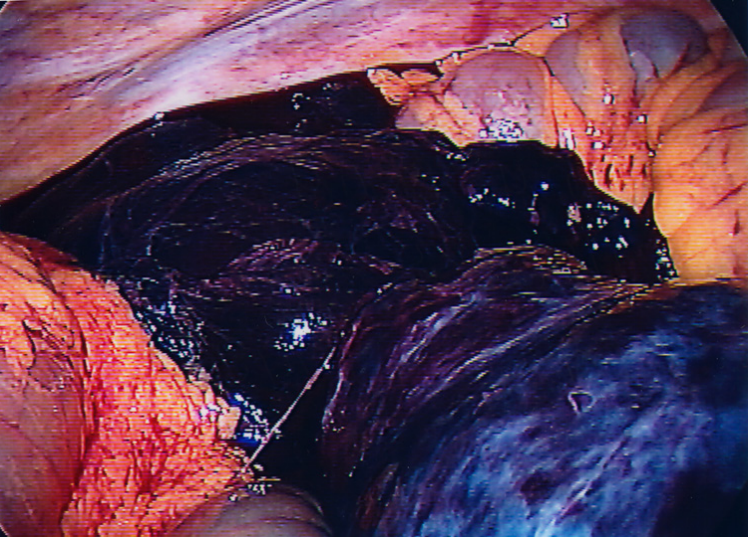
Initial view at laparoscopy showing hemorrhage from the cyst.

**Figure 2:**
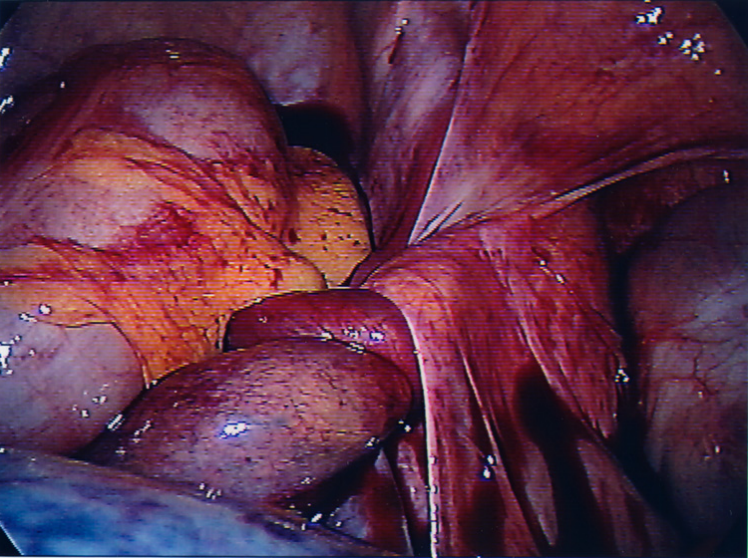
The torted right fallopian tube.

**Figure 3:**
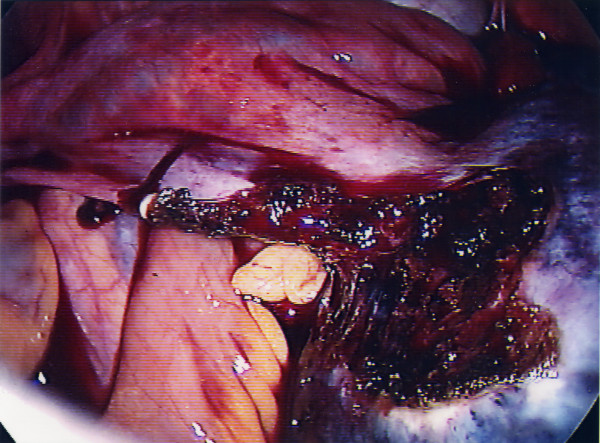
Dissection of the cyst wall from the residual ovarian tissue.

**Figure 4:**
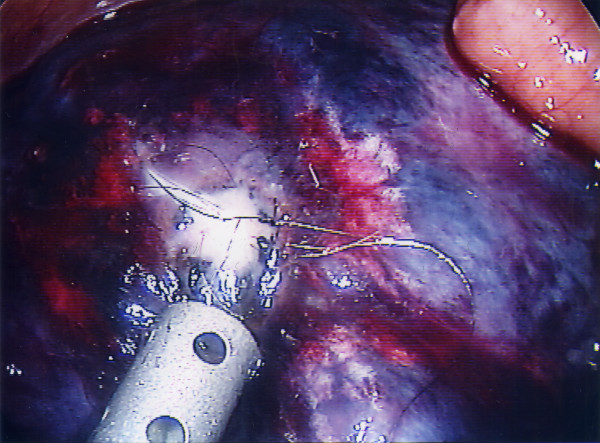
Controlled aspiration of the cyst.

**Figure 5:**
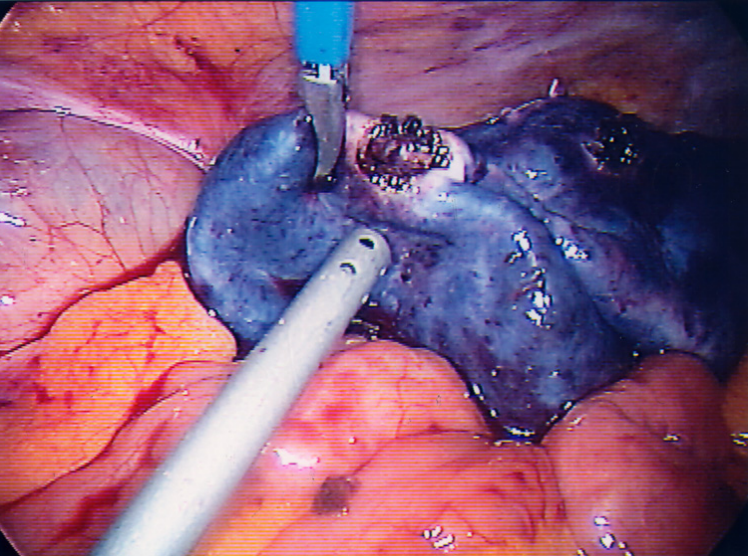
The collapsed cyst.

After induction of general anesthesia it became apparent that there was a large central abdominal mass. Laparoscopy using one 10 mm and two 5 mm ports showed bleeding from a large torted right ovarian cyst of approximately 15 cms diameter [figure 1, 2] and an enlarged left ovary. The cyst was untorted and the right fallopian tube was viable. The cyst was dissected free of the residual right ovary [figure 3] and its contents carefully aspirated [figure 4]. The collapsed cyst [figure 5] was then placed into a small bag and removed. Thorough and complete washout of the blood clot was done with normal saline. She made a very good recovery and was discharged the next day. Pathology showed a benign dermoid cyst. A subsequent pelvic ultrasound showed a simple cyst in the left ovary.

## Discussion

A dermoid is a benign, cystic lesion containing tissue from all three embryonic layers; endoderm, mesoderm and ectoderm. Ovarian dermoids constitute 10–15% of ovarian tumours and tend to occur in young women in the reproductive years, although presentation has been reported in prepubertal and elderly patients [1].

They are slow growing structures; one recent study found a growth rate of 1.8 mm/year in pre-menopausal women [2]. Ovarian dermoids present with discomfort, pain or pressure symptoms, or when a complication occurs. Torsion is the most common complication occurring in approximately 3.5% of cases [1]. Rupture with possible chemical peritonitis and subsequent granulomatous peritoneal deposits occurs in up to 2% of cases [3]. A dermoid cyst may rarely rupture into the bladder, presenting as recurrent urinary tract infections or more bizarrely as pilimiction. Rupture into the bowel with passage of dermoid structures per rectum has also been reported. Less than 1% of dermoid cysts are malignant [1].

The first laparoscopic cystectomy was performed in 1989 and since this time various spillage rates have been reported [4]. This is partly due to variations in the definition of 'spillage'; even purposeful puncture of the cyst is judged by some authors as 'spillage'. A recent study of 83 cases quoted a spillage rate, similar to that of other authors, at around 66% and this was associated with no cases of the chemical peritonitis [1]. Similarly, a study of 12 laparoscopic dermoid cyst resections, all of which were associated with intraoperative spillage and copious lavage, did not lead to a single case of chemical peritonitis [5]. However, there are reports of chemical peritonitis following rupture of a dermoid cyst during laparoscopy and also a report of intraperitoneal dissemination in a patient who underwent laparoscopic cystectomy for a dermoid that had undergone malignant transformation [6,7]. Of course, the cyst can always be placed into a retrieval bag, which can then be brought externally through a port site. The cyst can then be opened and the contents aspirated and this would avoid completely any risk of contamination

Laparoscopy is the preferred technique for diagnosing and treating the acute abdomen. In our case no preoperative scan was done and it was not until general anesthesia was induced that a mass was felt. Although in our case there was bleeding from the ruptured, ischemic cyst, the true cyst wall was intact. Unfortunately we did not have a large enough retrieval bag available on the day so a controlled aspiration of the cyst was done prior to placing it in a retrieval bag. No 'dermoidal contents' were spilt and the operation was completed with a thorough washout with normal saline. In our opinion there was no chance of chemical peritonitis from the procedure.

## Conclusion

Ovarian dermoid cysts may present as an emergency after torsion or rupture and from time to time general surgeons will encounter such a case. Laparoscopic management is beneficial and preservation of ovarian tissue and the fallopian tube is usually possible. We would recommend placing the cyst into a bag and externalizing it prior to aspiration to avoid any risk of contamination.
